# Incidence of Herpes Simplex Virus Keratitis in HIV/AIDS patients compared with the general population

**Published:** 2015

**Authors:** M Burcea, A Gheorghe, M Pop

**Affiliations:** * Clinical Emergency Eye Hospital, Bucharest, Romania

**Keywords:** herpes simplex virus, HIV/ AIDS, keratitis

## Abstract

Acquired immune deficiency syndrome (AIDS) is associated with a wide spectrum of systemic and ocular infectious diseases. Little information is known about Herpes Simplex Virus type 1 (HSV-1) keratoconjunctivitis in association with AIDS.

Because HSV-1 is becoming, day by day, a common eye disease (nearly 100% patients of over 60 years old harbor HSV in their trigeminal ganglia at autopsy), this article discussing a worldwide public health problem.

**Aim.** The purpose of this paper is to compare the incidence and clinical aspects of HSV-1 Keratitis in HIV/ AIDS patients compared with the general population who develops HSV- 1 Keratitis.

**Method.** The study is retrospective and comparative. Each patient was examined thoroughly at the biomicroscope ocular slit after corneal staining with fluorescein or rose bengal. Visual acuity, intraocular pressure and corneal sensitivity were also examined.

**Results.** From 170 patients with HIV and ocular anterior segment disorders, 47 patients had viral etiology. 58 patients had keratitis; 14 of them were HSV-1 keratitis.

**Conclusion.** Doctors should be aware of the existence of the ocular damage in HIV/ AIDS and emphasize the importance of regular ophthalmologic examination of patients with HIV/ AIDS as HSV infection is common nowadays among the general population.

**Abbreviations:** HSV = herpes simplex virus, HIV = human immunodeficiency virus, AIDS = acquired immunodeficiency syndrome

## Introduction

HIV infection produces a slow, progressive, specific degradation of immune, defensive mechanism, which explains why under a certain threshold of immune defense, patients contact opportunistic infections. This is the main cause of morbidity and ocular disease with the highest potential of destruction in patients with AIDS.

Each day, 6800 patients become HIV positive and 5700 of them die worldwide. Today there are around 17.819 reported cases of HIV/ AIDS in Romania.

Coinfection with HSV in common in patients with CD4 deficiency. The Herpesviridae family of double-stranded DNA viruses is an important cause of ocular infection. Direct contact with infected lesions or their secretions spread HSV infection but most commonly occurs as a result of exposure to viruses shed asymptomatically.

The primary ocular HSV infection typically manifests as a unilateral blepharoconjunctivitis. The conjunctival inflammatory response is follicular and accompanied by a palpable preauricular lymph node [**1**-**6**].

Clinic forms:

1. Epithelial keratitis with dendritic or geographic corneal ulcer 

2. Disciform keratitis with stromal and epitheliam oedema, keratic precipitates, folds in Descemet membrane, Wessely immune ring of stromal haze.

3. Necrotizing stromal keratitis

4. Neurotrophic ulceration with non-healing epithelial defect

## Methods

Because we have a good collaboration with the Ophthalmology Office of “Matei Bals” Infectious Disease Institute, we included in our study 170 HIV positive patients with anterior segment signs of ocular inflammation and infection.

The examination sheet of each patient registered the age, sex, number of CD4 copies /μL at presentation, adherence to the treatment. Detailed ocular examination displayed visual acuity measurement, intraocular pressure measurement, examination of the anterior segment and posterior segment of the eye, staining of the cornea with fluorescein or rose bengal.

These individual data were analyzed and results were reported in percentage or absolute numerical value.

We compared the HIV population with general population who presented in the Ophthalmology Emergency Room, over a year, with photophobia, epiphora and signs of ocular inflammation.

## Results

From 170 patients with HIV and ocular anterior segment disorders, 47 had a viral etiology. Therefore, 28 % of the patients had a viral implication.

From 170 patients with HIV and ocular anterior segment disorders, 58 patients had keratitis. In addition, 34% developed keratitis. From 58 keratitis, 14 were viral. Therefore, 24% of the HIV positive patients with anterior segment involvement had viral (HSV 1) keratitis. The evolution to vascularized leucoma was seen in half (50%) of the cases with HSV keratitis. We observed the predilection for peripheral versus central involvement and a median healing time of 3 weeks by using topical antiviral therapy.

**Table 1:** Anterior Segment Ocular manifestations

Anterior segment ocular disorders	Number of patients
Viral disorders	47
Keratitis	58
Viral keratitis ( HSV-1)	14

10000 patients were examined in the Emergency Room over a year period of time. The incidence of HSV-1 keratitis in the general population was of 357 cases. Therefore, the incidence was of 3.57%. The most frequent types were dendritic keratitis; approximately 202 cases were reported. In addition, the incidence of dendritic keratitis was of 56.3%. Stromal keratitis was reported in 123 cases, so the incidence was of 29% and geographic keratitis was reported in 32 cases, so the incidence was of 9% [**7**].

**Table 2:** Incidence of HSV-1 keratitis in the general population (10000 cases)

Type of HSV-1 keratitis	Number of patients
General HSV-1 keratitis	357
Dendritic keratitis	202
Stromal keratitis	123
Geographic keratitis)	32

In the general population, the median healing time was of 2 weeks with topical antiviral therapy.

**Table 3:** Median Healing time

Patients HIV positive	3 weeks
General population	2 weeks

## Conclusions

Herpetic keratitis remains an epidemiologically important eye disease that justifies the need to pursue health and research programs aimed at improving the outcome of ocular herpetic disease.

Herpetic keratitis in HIV positive patients has a higher rate of incidence and recurrence. This is why these patients should perform ophthalmologic consults at every 6 months.
